# Influence of the milling parameters on the nucleophilic substitution reaction of activated β-cyclodextrins

**DOI:** 10.3762/bjoc.13.184

**Published:** 2017-09-07

**Authors:** László Jicsinszky, Kata Tuza, Giancarlo Cravotto, Andrea Porcheddu, Francesco Delogu, Evelina Colacino

**Affiliations:** 1 Dipartimento di Scienza e Tecnologia del Farmaco, University of Turin, via P. Giuria 9, 10125 Turin, Italy; 2 Cyclolab Cyclodextrin R&D Laboratory, Ltd., Illatos út 7, 1192 Budapest, Hungary; 3 Dipartimento di Scienze Chimiche e Geologiche, Università degli Studi di Cagliari, Cittadella Universitaria, SS 554 bivio per Sestu, 09028 Monserrato (Ca), Italy; 4 Dipartimento di Ingegneria Meccanica, Chimica, e dei Materiali, Università degli Studi di Cagliari, via Marengo 2, 09123 Cagliari, Italy; 5 Université de Montpellier, Institut des Biomolécules Max Mousseron (IBMM) UMR5247 CNRS-UM-ENSCM, Université de Montpellier, cc1703, Place Eugène Bataillon, 34095 Montpellier Cedex 05, France

**Keywords:** cyclodextrins, milling parameters, nucleophilic substitution, planetary ball mill

## Abstract

The present work focuses on the mechanochemical preparation of industrially important β-cyclodextrin (CD) derivatives. Activated CDs have been reacted with nitrogen and sulfur nucleophiles using a planetary mill equipped with stainless steel, zirconia and glass milling tools of different sizes. It is shown that the milling frequency and the number as well as the size of the milling balls have an effect on the nucleophilic reaction.

## Introduction

Their hollow structures make cyclodextrins (CDs) a class of carbohydrates that can form inclusion complexes with organic molecules, inorganic salts and complex metal ions [1]. Such a unique capacity makes CD derivatives crucial in a number of every-day sectors, ranging from paintings [2] to food [3]. The availability of convenient methods for their large-scale production has made CDs all but ubiquitous, including their use in a variety of investigations at the cutting edge of biological [4] and chemical science research [5]. However, there is still considerable room for the synthesis of specific CDs on the laboratory scale. This is the case, for instance, with 6^I^-monoamino-6^I^-monodeoxy-β-CD, which is easily prepared via the reduction of the parent mono-azido derivative and is used in analytical chemistry as chiral stationary phase [6]. CDs functionalized with triazole substituents can be similarly prepared through click reactions involving the azido group as a dipolarophile [7], and utilized as suitable starting material to access hydroxy functionality after derivatization [8]. Although the preparation of carbohydrate-based complexes in a ball mill has been already reported [9–11], the use of mechanical activation for the chemical derivatization of CDs has been rather sporadic [12–15]. In this respect, it is worth noting that CDs exhibit a characteristic reactivity profile. Neither traditional synthetic routes nor a conventional carbohydrate activation methodology allow for CD derivatization. The major issues stem from the differing solubility of the reagents in organic solvents, meaning that high boiling polar solvents, such as DMF or DMSO, need to be used. However, these solvents are difficult to remove and usually have considerable energy contribution. Under these circumstances, the promise shown by the mechanical processing of solids of enabling chemical transformations in the absence of solvent phases renders mechanical activation extremely appealing. This is particularly true in light of the well-known capability of mechanical treatment to induce significant enhancements in chemical reactivity.

Despite the vast amount of literature on the mechanically activated synthesis of organic molecules [16–23], CD mechanochemistry offers significant challenges. For instance, the molecular weight negatively affects the reaction design and is almost one order of magnitude higher here than for common organic molecules. The laborious preparation of the starting CD-tosylate [24–25], and the considerable reactant molecular mass differences are also elements of complexity. The mechanical processing of CDs in the absence of solvent therefore promises to simplify the work-up and allows the almost complete utilization of the CD key-intermediate [13], in comparison with the classic method [6]. Moreover, the absence of a solvent, high-boiling-point ones in particular, could prevent the undesired side-reactions, that would be caused by the decomposition of DMF (formation of dimethylamine), by hydrolysis (from residual crystal water), and by alkylation and/or oxidation (DMSO) [13], leading to cleaner reaction profiles under mechanochemical conditions. Previous work on mechanically activated substitutions on tosyl ester-activated CDs resulted in high yields of the targeted 6-monoderivatized CDs, but also in complex isolation procedures due to the large number of small balls used (50 of ø 5 mm + 1500 of ø 1 mm steel balls) [13]. Despite the longer milling times, using less balls allow outcomes to be improved [14]. This work takes the above-mentioned results as a base from which to address the mechanochemical synthesis of 6^I^-monoazido-6^I^-monodeoxy-β-CD and 6^I^-*S*-monodeoxy-6^I^-monothiouronium-β-CD tosylate (TU-β-CD), an important CD intermediate for the preparation of 6^I^-*S*-monodeoxy-6^I^-monothio-β-CD [26]. Having selected the 6^I^-*O*-monotosyl-β-CD (Ts-β-CD) as the benchmark, the nucleophilic displacement of the tosylate group in the presence of azido or thiourea (TU) nucleophiles was chosen for the study under different milling conditions. The reaction was performed in a planetary ball mill and the processing parameters were systematically varied with the aim of pointing out their influence on the nucleophilic substitution reactions in terms of rate and yield. Specifically systematic variation involved rotation speed, milling tool materials, ball number and size, ball-to-powder mass ratio, the fraction of reactor volume occupied by balls and the reactor volume itself.

## Results and Discussion

**Scheme 1:**
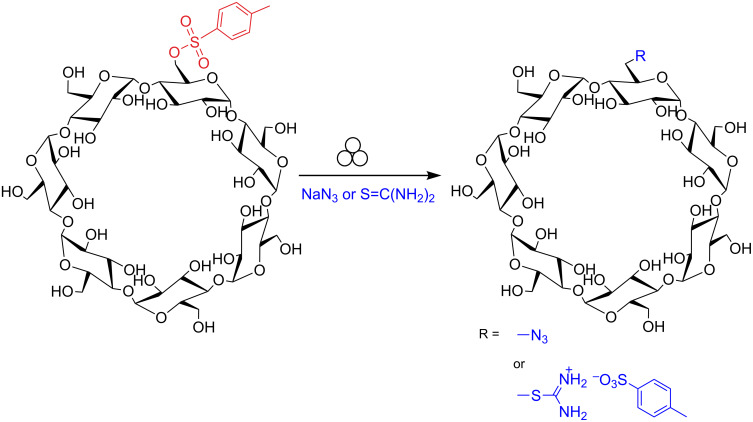
Nucleophilic substitution of the 4-toluenesulfonyl group. The formalism for the mechanochemical activation was suggested by Rightmire [27].

We previously reported [13] a successful scale-up monoazidation reaction of Ts-β-CD (the reaction scale was 6.5 g, 5 mmol) in a ball-mill (Supporting Information File 1, Table S1, entries 1–4). Considering that the preparation of Ts-β-CD is laborious [24–25], its commercial availability is restricted by high costs and limited number of producers, the systematic investigation on the influence of the milling parameters on the reaction outcome was investigated using a reaction scale of dominantly 1 mmol of substrate, in the presence of 3 equivalents of NaN_3_ or thiourea (TU) as nucleophiles (Scheme 1). Being the removal of the starting Ts-β-CD from the 6-monoazido-β-CD complicated due to the solubility similarities, the time to reach complete conversion (> 99.5%, defined as milling time) of the starting material had been targeted as main control parameter (see details in Supporting Information File 1).

No significant role can be ascribed to the temperature, since systematic measurements under different processing conditions indicated that it never exceeded 72 °C. Further, no degradation of the activated Ts-β-CD was observed.

The yield of the mechanically induced azidation is invariably higher than the one observed in our previous work [13]. However, the rate of the reaction involving the more nucleophilic TU is considerably lower. Chemical conversion data regarding the reactions performed under different milling conditions are summarized in Table S1 (Supporting Information File 1). It can be seen that the reaction yield shows significant scatter. No definite relationship between the set of processing parameters and the yield can be identified. Nevertheless, sets of balls with different size seemingly assure the best performances in terms of yield and reaction rate, enabling full substrate conversion in shorter reaction times (Supporting Information File 1, Table S1, entries 2, 6, 11, and 12).

The observed yield enhancement can be tentatively related to the effectiveness of energy transfer, which can be expected to increase as the volume occupied by balls inside the reactor increases, thus allowing milling conditions to approach frictional regimes.

**Figure 1:**
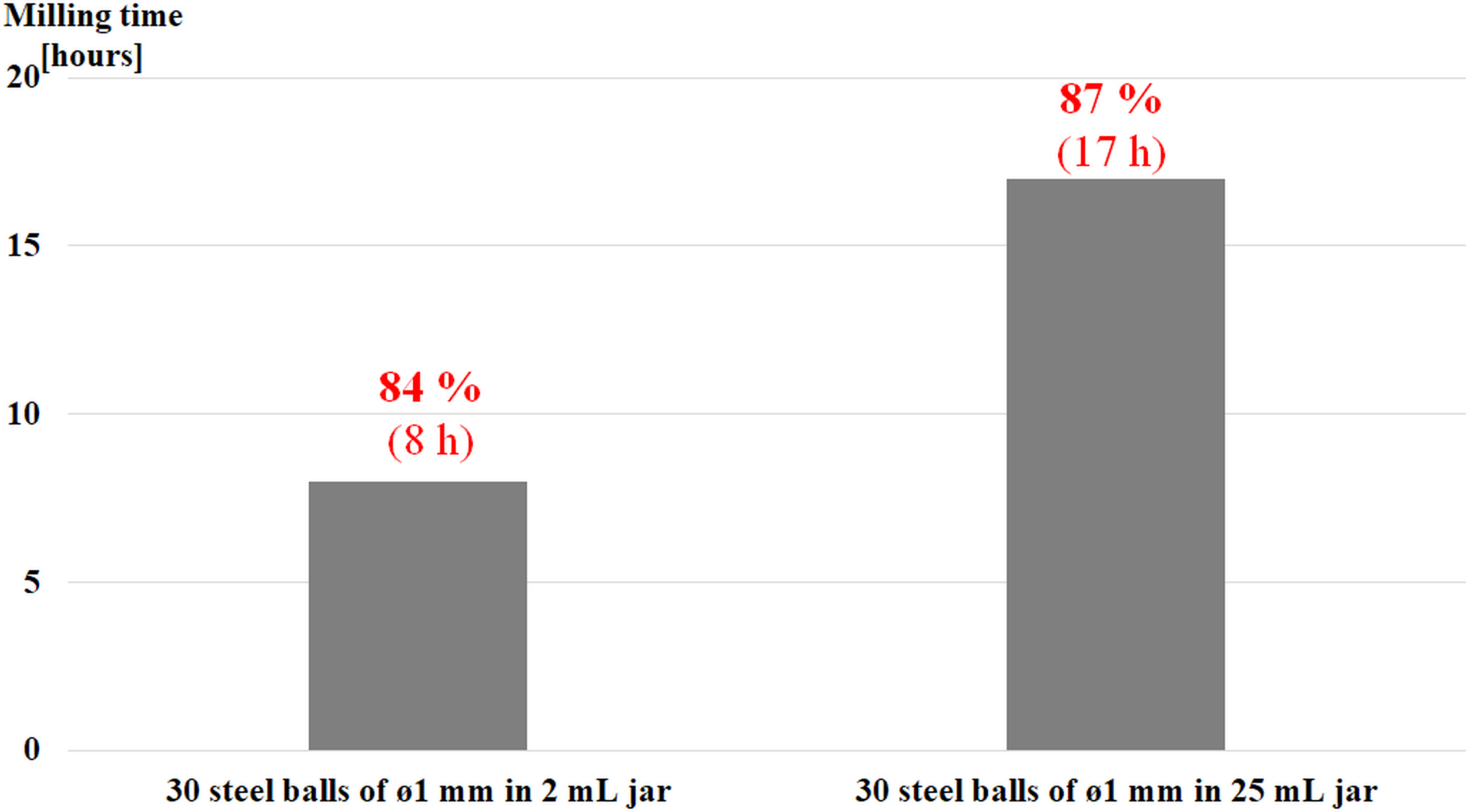
Effect of jar size on the reaction time using an equal number (30) of steel balls (ø 1 mm) for the Ts → N_3_ exchange reaction in glass vials at 550 min^−1^ sun wheel speed.

In the attempt of clarifying the role of the volume fraction occupied by balls inside the reactor, the nucleophilic substitution with NaN_3_ was performed using glass reactors 2 and 25 mL in volume and the same number of balls of equal size (30 balls of 1 mm in diameter). The experimental findings are summarized in Figure 1 and Supporting Information File 1, Table S1 entries 18 and 19. The reaction rate definitely increases as the volume fraction occupied by balls inside the reactor increases. Therefore, it would appear that an increasing ball contact density shortens milling time.

**Figure 2:**
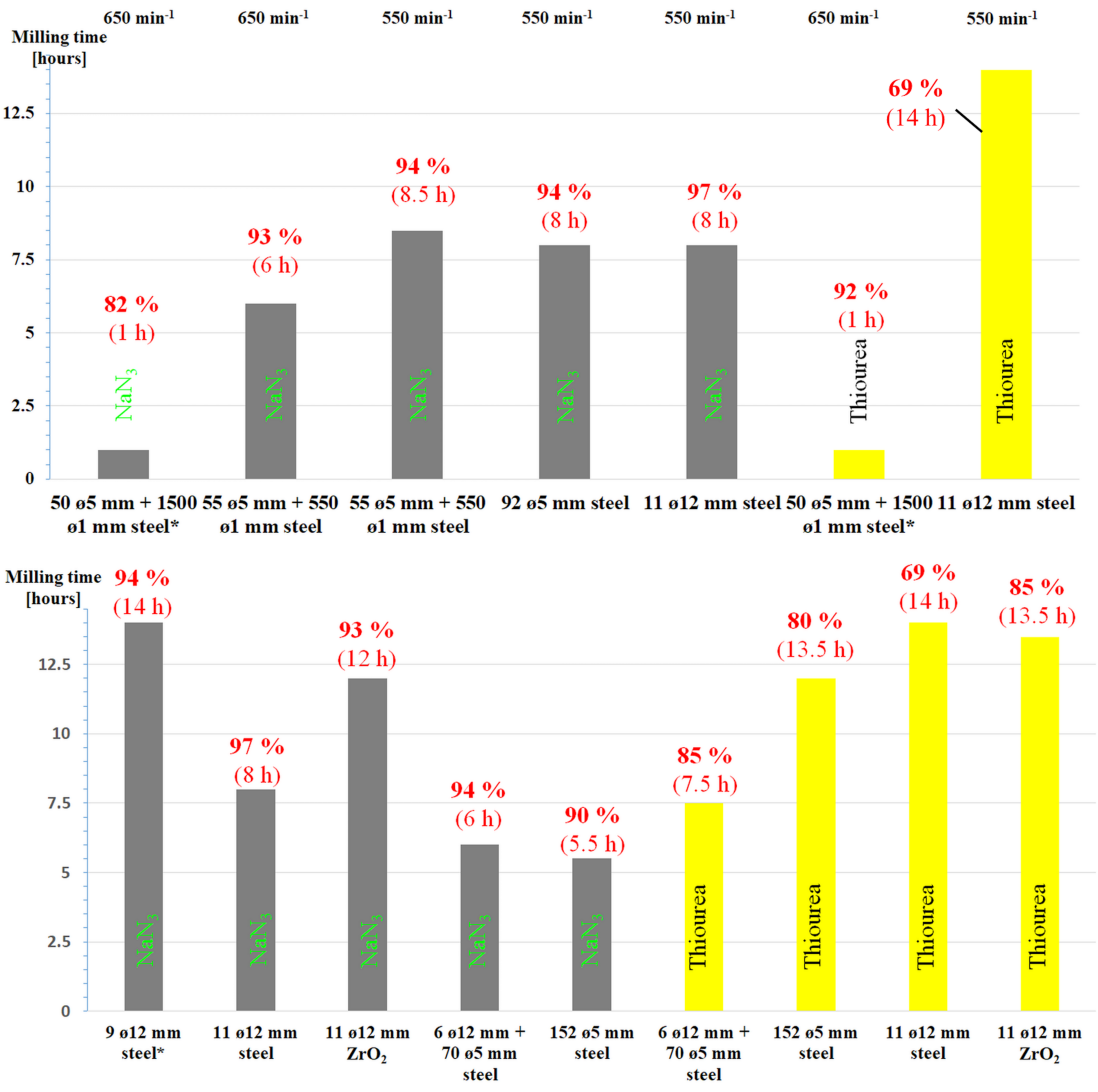
Effect of ball size on the reaction time to a full conversion of Ts-β-CD: a) reactions performed at constant total steel ball weight of ca. 45 g (*weight of steel balls ca*.* 70 g for comparison with [13]); b) the number (and size) of balls were combined to be equal to the volume occupied by 11 balls of ø 12 mm (ca. 10 mL) at 550 min^−1^ (*weight of steel balls ca. 40 g kept similar to 11 zirconia balls of 12 mm in diameter (ø) for comparison). Values given on the graph bars indicate, respectively, the yield and the reaction time to achieve full conversion of the starting Ts-β-CD.

Further support for the hypothesis that the higher number of impacts among balls per unit of time enhances the outcome of the reaction comes from data shown in Figure 2a and Supporting Information File 1, Table S1 entries 6 and 7. The data in Figure 2 refer to experiments performed varying the ball size while keeping the total volume occupied by balls approximately constant. Under these circumstances, the number of contacts between balls increases as the ball size decreases. Based on the above-mentioned hypothesis, reaction rate should be expected to increase. In line with expectations [18], the experimental findings indicate that the smaller the ball size, the shorter the reaction time for both nucleophiles.

**Figure 3:**
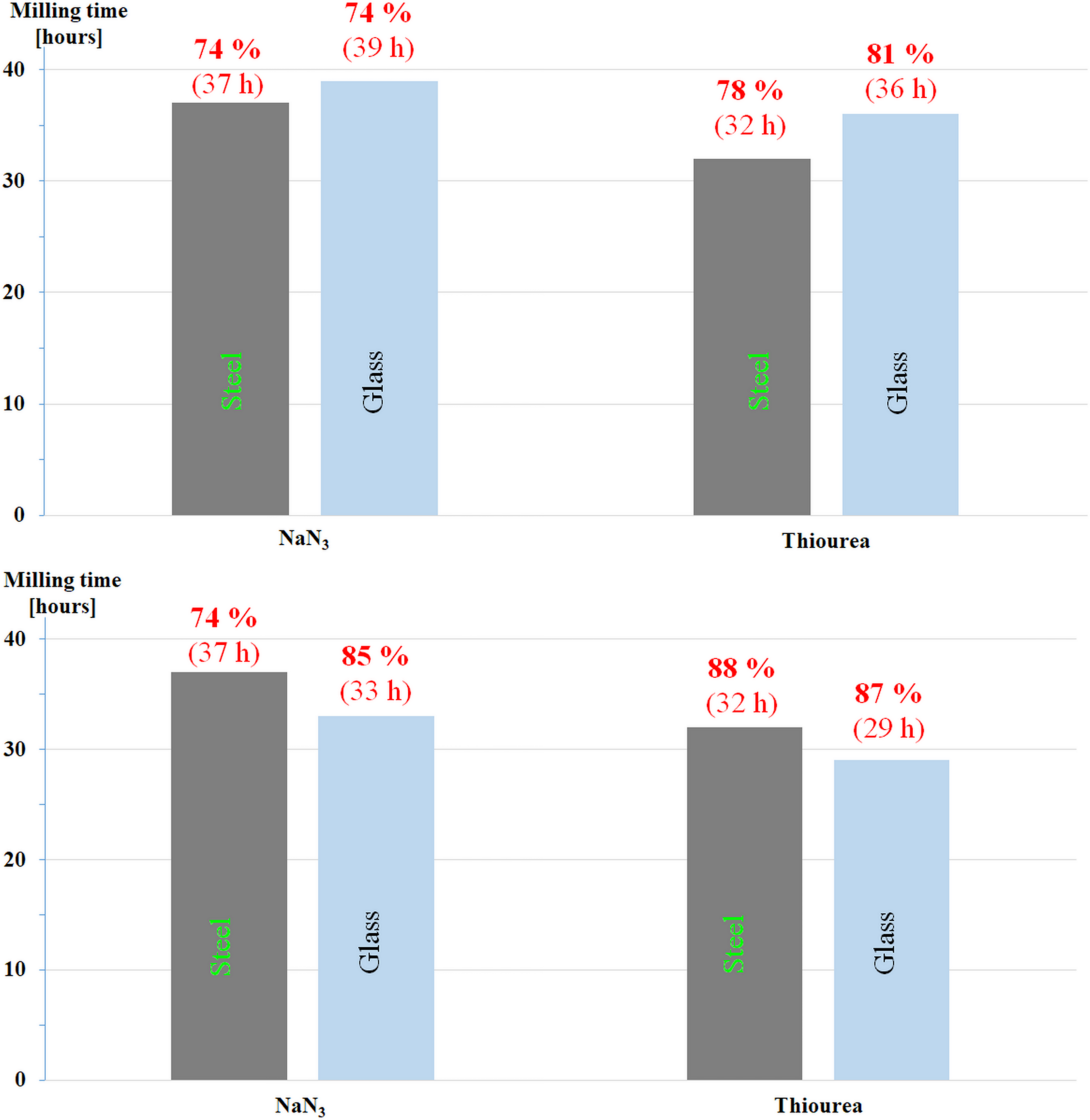
Reaction time as a function of ball materials at 550 min^−1^ in glass vials of 25 mL: a) equal weight: 60 steel balls of ø 1 mm (1.8 g) vs*.* 20 glass balls of ø 3 mm (1.8 g); b) 60 steel balls of ø 1 mm (*m*_B_ = 1.8 g, *m*_B_/*m*_R_ ca. 12, Φ_MB,packing_ = 0.003) vs. 60 glass balls of ø 3 mm (*m*_B_ = 5.4 g, *m*_B_/*m*_R_ ca. 35, Φ_MB,packing_ = 0.077). Values given on the graphic bars indicate, respectively, the yield and the reaction time to achieve full conversion of the starting Ts-β-CD.

Most experiments were performed at a planetary mill sun wheel speed of 550 min^−1^. Under these conditions, the reaction involving NaN_3_ as the nucleophile was investigated using the same weight of balls (ca. 45 g), but varying the ratio of balls with different size. The data in Figure 2a and Supporting Information File 1, Table S1 entries 6–8 and 13 show no dramatic change in reaction rate. TU exhibits a slower kinetics than NaN_3_ under the same milling conditions (Supporting Information File 1, Table S1, entry 13 vs. 16), which hints at substrate-dependent reactivity (Figure 3b and Supporting Information File 1, Table S1, entries 9, 11, 13 and 14 for NaN_3_ vs*.* 10, 12, 16 and 17 for TU, respectively).

However, from the experiments the highest sun wheel speed at 650 min^−1^ resulted in faster reaction (Figure 2a) and the number of balls seemed to have less influence on the investigated reaction. It is assumed that a combination of the kinetic energies of the individual balls and the number of impacts can play an important role in the reaction rate.

The material constituting milling tools affects the outcomes of the substitution reaction. Data in Figure 2b (Supporting Information File 1, Table S1, entries 13 and 14 vs. 16 and 17, respectively) shows that, as far as NaN_3_ was used in combination with 12 mm balls, the best reaction yield and rate were obtained in stainless steel reactors (Supporting Information File 1, Table S1, entries 13 and 14). By contrast, under the same processing conditions, ZrO_2_ gave the best performances in reactions involving TU (Supporting Information File 1, Table S1, entries 16 and 17). Thus, NaN_3_ seemingly displayed stronger nucleophilicity than TU when stainless steel milling tools were utilized and vice versa for ZrO_2_ milling tools.

In another set of experiments, for the same nucleophile, comparative experiments were performed using a total number of glass balls having the same weight (1.8 g) of 60 steel balls of 1 mm ø (Figure 3a and Supporting Information File 1, Table S1, entries 20/22 for NaN_3_ and 21/23 for TU).

The less hard glass balls (and jars) are in general less effective in terms of energy transfer as compared to steel. This was confirmed in the case of TU (Figure 3a and Supporting Information File 1, Table S1 entries 21 vs. 23), while milling times did not considerably change as expected [17] in the case of NaN_3_ (Figure 3a and Supporting Information File 1, Table S1 entry 20 vs. 22). However, an increase of the number of glass balls, led to somehow better yields after slightly shorter reaction times for both nucleophiles (Figure 3a vs. 3b), even at an improved *m*_B_/*m*_R_ ratio and Φ_MB,packing_ values (Figure 3b and Supporting Information File 1, Table S1, entries 20, 21 vs. 24, 25).

Finally, the experimental findings collected in Supporting Information File 1, Table S1 show that for a larger volume occupied by balls inside the reactor, faster reactions were observed, independent of the material that the milling tools were made from.

## Conclusion

Mechanical activation in a planetary ball mill allows the studied reactions to take place at a rate higher than the corresponding reactions in solution. Indeed, the nucleophilic substitution of tosyl groups is very slow at *T* < 80 °C (in DMF), while in water (at 50–70 °C) the most competitive side reaction is the hydrolysis of the starting material. Moreover, mechanochemical activation allowed solve one of the major problems for cyclodextrin derivatization in solution. This is usually related to the very different solubilities of the reagents, thus requiring energy transfer by heating to induce reactions. Although it is difficult to reach a compromise between the reaction and side reactions, without a massive energy transfer the derivatizations are rarely successful in solution. By mechanochemistry, the reactivity is mainly affected by the sun wheel speed and the number and size of balls for both nitrogen and sulfur nucleophiles. In general, reaction rates reach a maximum as the volume fraction occupied by balls inside the reactor increases and the ball size decreases but no simple correlation was found. Consequently, it seems reasonable to connect reaction yield and rate with the total number of contact between balls. Unlike the reactivity in solution, under mechanochemical conditions the sulfur nucleophile (thiourea, TU) was less effective than the azide ion in the substitution reaction. A similar reversal of reactivity has been already observed for halogens [12–13,28]. The experimental findings lend support to the idea that mechanical activation can induce chemical reactivity [29] and selectivity [30] which is different to that observed in solution, which can be further complicated by the inclusion complex formation property of cyclodextrins.

How exactly the milling parameters influence the kinetics and the mechanisms of organic reactions is still question of investigation in the scientific community. Even though our contribution tries to delineate some trends, additional investigations and experiments need to be performed for a fully understanding of this still understudied and poorly understood aspect of mechanochemistry.

## Supporting Information

File 1 Experimental procedures and technical details.

## References

[1] Szejtli J, Osa T (1996). Cyclodextrins. Comprehensive Supramolecular Chemistry.

[2] Hashidzume A, Takashima Y, Yamaguchi H, Harada A, Atwood J L (2017). Cyclodextrin. Comprehensive Supramolecular Chemistry II.

[3] Martina K, Binello A, Lawson D, Jicsinszky L, Cravotto G (2013). Curr Nutr Food Sci.

[4] Adeoye O, Cabral-Marques H Int J Pharm.

[5] Zhu G, Yi Y, Chen J (2016). TrAC, Trends Anal Chem.

[6] Jicsinszky L, Iványi R (2001). Carbohydr Polym.

[7] Earla A, Braslau R (2014). Macromol Rapid Commun.

[8] Jicsinszky L, Ivanyi R Proceedings of the 10th International Symposium on Cyclodextrins.

[9] Rinaldi L, Binello A, Stolle A, Curini M, Cravotto G (2015). Steroids.

[10] Hedges A, Tenbarge F (1991). Cyclodextrin complexing method. U.S. Pat. Appl..

[11] Czugler M, Pintér I (2011). Carbohydr Res.

[12] Jicsinszky L, Caporaso M, Martina K, Gaudino E C, Cravotto G (2016). Beilstein J Org Chem.

[13] Jicsinszky L, Caporaso M, Tuza K, Martina K, Gaudino E C, Cravotto G (2016). ACS Sustainable Chem Eng.

[14] Jicsinszky L, Caporaso M, Gaudino E C, Giovannoli C, Cravotto G (2017). Molecules.

[15] Menuel S, Doumert B, Saitzek S, Ponchel A, Delevoye L, Monflier E, Hapiot F (2015). J Org Chem.

[16] Sánchez-Jiménez P E, Valverde J M, Perejón A, de la Calle A, Medina S, Pérez-Maqueda L A (2016). Cryst Growth Des.

[17] Stolle A, Szuppa T, Leonhardt S E S, Ondruschka B (2011). Chem Soc Rev.

[18] Stolle A, Schmidt R, Jacob K (2014). Faraday Discuss.

[19] Ranu B, Stolle A (2015). Ball Milling Towards Green Synthesis: Applications, Projects, Challenges. RSC Green Chemistry.

[20] Wang G-W (2013). Chem Soc Rev.

[21] James S L, Adams C J, Bolm C, Braga D, Collier P, Friščić T, Grepioni F, Harris K D M, Hyett G, Jones W (2012). Chem Soc Rev.

[22] Tan D, Loots L, Friščić T (2016). Chem Commun.

[23] Do J-L, Friščić T (2017). ACS Cent Sci.

[24] Law H, Benito J M, Garcia Fernandez J M, Jicsinszky L, Crouzy S, Defaye J (2011). J Phys Chem B.

[25] Brady B, Lynam N, O’Sullivan T, Ahern C, Darcy R (2000). Org Synth.

[26] Liu A, Zhao Q, Guan X (2010). Anal Chim Acta.

[27] Rightmire N R, Hanusa T P (2016). Dalton Trans.

[28] Konnert L, Dimassi M, Gonnet L, Lamaty F, Martinez J, Colacino E (2016). RSC Adv.

[29] Konnert L, Reneaud B, de Figueiredo R M, Campagne J-M, Lamaty F, Martinez J, Colacino E (2014). J Org Chem.

[30] Hernández J G, Bolm C (2017). J Org Chem.

