# Supplementary material for: Influence of the milling parameters on the nucleophilic substitution reaction of activated β-cyclodextrins
Source: Beilstein J Org Chem. 2017 Sep 7;13:1893–9. doi: 10.3762/bjoc.13.184 (PMC5629382; doi:10.3762/bjoc.13.184)
Supplement: File 1 — Experimental procedures and technical details. [file Beilstein_J_Org_Chem-13-1893-s001.pdf]

Supporting Information
for
Influence of the milling parameters on the nucleophilic substitution reaction of activated β-cyclodextrins

László Jicsinszky*[1], Kata Tuza[2], Giancarlo Cravotto[1], Andrea Porcheddu[3], Francesco Delogu[4] and Evelina Colacino*[1,5]

Address: [1]Dipartimento di Scienza e Tecnologia del Farmaco, University of Turin, via P. Giuria 9, Turin, 10125, Italy, [2]Cyclolab Cyclodextrin R&D Laboratory, Ltd., Illatos út 7, Budapest, 1192, Hungary, [3]Dipartimento di Scienze Chimiche e Geologiche, Università degli Studi di Cagliari, Cittadella Universitaria, SS 554 bivio per Sestu, 09028 Monserrato (Ca), Italy, [4]Dipartimento di Ingegneria Meccanica, Chimica, e dei Materiali, Università degli Studi di Cagliari, via Marengo 2, 09123 Cagliari, Italy and [5]Université de Montpellier, Institut des Biomolécules Max Mousseron (IBMM) UMR5247 CNRS-UM-ENSCM, Université de Montpellier, cc1703, Place Eugène Bataillon, 34095 Montpellier Cedex 05, France

Email: László Jicsinszky - ljicsinszky@gmail.com; Evelina Colacino - evelina.colacino@umontpellier.fr

*Corresponding author

Experimental procedures and technical details

Table of Contents

Table S1: Processing parameters and reaction yield (the sun wheel speed was: 650 min^{-1} for entries 1–6, 550 min^{-1} for entries 7–25)	S3
Experimental section	S4
Figure S1: 25 mL glass jar milled in a planetary system using a 500 mL stainless steel jar as host cavity**.**	S4
General experimental procedure on a 0.001 mol scale (Table S1, entries 2–4 and 6–17).	S6
General experimental procedure on 0.0001 mol scale (Table 1, entries 1, 5, 18–25).	S7
References	S7

Table S1: Processing parameters and reaction yield (the sun wheel speed was: 650 min^{-1} for entries 1–6, 550 min^{-1} for entries 7–25).

Entry	Reagent	V_{jar}[a,b] [mL]	Ball ø[a,b] [mm]	Number of balls[b]	ΣV_{ball}[c] [mL]	m_B[d] [g]	m_B/m_R[d]	$\Phi_{MB,packing}$[e]	Milling time [hours]	Yield[f] [%]
1	NaN_3	50	5+1	50+1500	4.1	69.5	463.3	0.128	1	82[g,h]
2	NaN_3	50	5+1	50+1500	4.1	69.5	46.8	0.128	1	69[g]
3	NaN_3	50	5+1	50+1500	4.1	69.5	18.6	0.128	1	62[g]
4	NaN_3	50	5+1	50+1500	4.1	69.5	10.2	0.128	1.5	90[g]
5	TU	50	5+1	50+1500	4.1	69.5	454.8	0.128	1	92[g,h]
6	NaN_3	50	5+1	55+550	3.9	43.5	30.2	0.122	6	93
7	NaN_3	50	5+1	55+550	3.9	43.5	30.2	0.122	8.5	94
8	NaN_3	50	5	92	6.0	45.1	30.3	0.188	8	94
9	NaN_3	50	5	152	9.9	74.4	50.1	0.311	5.5	90
10	TU	50	5	152	9.9	74.4	49.0	0.311	12	80
11	NaN_3	50	12+5	6+70[i]	10	58.2	39.2	0.309	6	94
12	TU	50	12+5	6+70[i]	10	58.2	38.3	0.309	7.5	85
13	NaN_3	50	12	11	9.9	43.9	29.5	0.303	8	97
**14**	**NaN_3**	**50**	**12**	**11**	**9.9**	**36.6**	**24.6**	**0.303**	**12**	**93**
15	NaN_3	50	12	9	8.1	35.8	24.1	0.248	14	94
16	TU	50	12	11	9.9	43.9	28.9	0.303	14	69
**17**	**TU**	**50**	**12**	**11**	**9.9**	**36.6**	**24.1**	**0.303**	**13.5**	**85**
18	NaN_3	*2*	1	30	0.01	0.9	5.8	0.021	8	84[h]
19	NaN_3	*25*	1	30	0.01	0.9	5.8	0.002	17	87[h]
20	NaN_3	*25*	1	60	0.08	1.8	11.9	0.003	37	74[h]
21	TU	*25*	1	60	0.08	1.8	11.8	0.003	32	88[h]
22	*NaN_3*	*25*	*3*	*20*	*0.28*	*1.8*	*12.0*	*0.026*	*39*	*78*[h]
23	*TU*	*25*	*3*	*20*	*0.28*	*1.8*	*11.8*	*0.026*	*36*	*81*[h]
24	*NaN_3*	*25*	*3*	*60*	*0.85*	*5.4*	*36.1*	*0.077*	*33*	*85*[h]
25	*TU*	*25*	*3*	*60*	*0.85*	*5.4*	*35.3*	*0.077*	*29*	*87*[h]

[a]Nominal value from the producer; [b]font style: normal - stainless steel, **bold - zirconia**, *italic - glass*; [c]the volume occupied by the balls was calculated from the equation $V = 4/3\,\pi\, r^3$; [d]m_B: weight of balls, m_R: total weight of reagents; [e]$\Phi_{MB,packing} = V_{Balls,packing}/V_{jar}$ with $V_{Balls,packing} = V_{calculated}/0.64$ [1,2]; [f]isolated yields. On millimolar scale, both sampling and filtration/material transfer losses during work-up may considerably affect the isolated yields (10 mg differences cause ca. 1% difference in yields). [g]from [4]; [h]0.1 mmol scale; [i]*ca.* 1:10 ratio, in comparison with entries 6 and 7.

Experimental section

β-Cyclodextrin hydrate was a generous gift from Roquette Frères (Lestrem, France). All other reagents were purchased from Alfa Aesar (Karlsruhe, Germany) and used without any further purification. 6^{I}-*O*-Monotosyl-β-CD (Ts-β-CD) was prepared and purified as described in [3] then dried as described in [4], and weights in the description were corrected with the water content (5%).

Ball mill: Retsch PM100 High Speed Planetary Ball Mill, reactions were performed: i) in a stainless steel or zirconium oxide jar of 50 mL (internal ø 43 mm), the reaction scale was 0.001 mol; ii) in glass vials 2 mL (internal ø 9 mm) and 25 mL (internal ø 24 mm), the reaction scale was 0.0001 mol. Sun wheel speed was 550 or 650 min^{-1}, the rotation direction was changed every 15 min, with a standby of 15 s in between each milling cycle. Ball size and materials: stainless steel (ø 1, 5 and 12 mm), zirconium oxide (ø 12 mm), and glass (ø 3 mm). The experiments performed in the glass vials were placed into a 500 mL stainless steel jar and fixed over a polystyrene auxiliary, to tightly keep the vial in the center of jar (Figure S1).

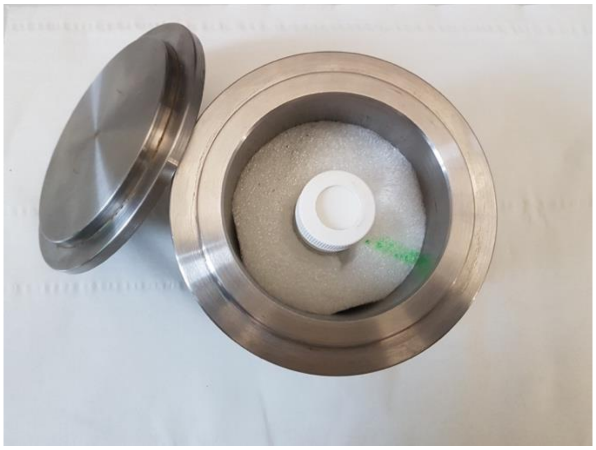

Figure S1: 25 mL glass jar milled in a planetary system using a 500 mL stainless steel jar as host cavity.

Thermometer: Lafayette TRI-88 no-contact thermometer with built-in laser pointer, with ±2 °C reading accuracy, distance to spot size = 8:1, measuring distance 18–23 cm. The measurement matrix formed “a five on a die”, two measurements were made at each point and the values were averaged.

TLC: Semiquantitative TLC experiments used Merck 5554 Silica gel 60, a saturated chamber, the regular running distance was 7 cm. Accurately weighed 10 mg (0.001 mol scale) or 5 mg (0.0001 mol scale) samples were dissolved in 50% EtOH to 2% concentration and 5 µL were dropped onto the TLC plate. At high Ts-β-CD content the samples were inhomogeneous, slowly sedimenting suspensions. Runs were performed in a saturated chamber with 10:7 (*v*/*v*) 1,4-dioxane:aq. NH_3 (28–30%) solvent system. The completely dry and NH_3-free TLC plate was sprayed with 20% cc. H_2SO_4/EtOH and heated for 10 min at 100–105 °C. Organic materials, except tosylate and thiourea, displayed as black spots. 6^I-*O*-Monotosyl-β-CD, 6^I-*S*-monodeoxy-6^I-monothiouronium-β-CD tosylate, tosylate and thiourea show strong UV absorption at 254 nm, 6^I-monoazido-6^I-monodeoxy-β-CD shows some UV absorption only, β-CD and sodium azide have no noticeable UV absorption at the given wavelength. Charring sensitivity of Ts-β-CD, β-CD and TU-β-CD under the used conditions is <0.25 µg. All the reactions were conducted until complete disappearance of Ts-β-CD (~ <0.5–0.25%). The spectral data of the final compounds were identical to those already reported in the literature [2]. The azido-β-CD samples were free of sodium tosylate and the TU-β-CD samples were free of thiourea and were pure enough to be used for further reactions.

General experimental procedure on a 0.001 mol scale (Table S1, entries 2–4 and 6–17).

Ts-β-CD (1.29 g, 0.001 mol) and NaN_3 (0.197 g, 0.003 mol) or thiourea (TU, 0.228 g, 0.003 mol) were mixed with a spatula, added to the jar containing the balls, then milled until complete disappearance of Ts-β-CD (~<0.5–0.25%). Sampling was after 60/90/180/360/420/etc. min. 'Bulk' temperatures at the end of the reaction were measured: 66–72 °C range for NaN_3 and 64–67 °C range for TU. The milling conditions (jar material, number and size of the balls, rotation speed and milling time) are detailed in Table S1 and in the general experimental section above.

Work-up and purification. As the reaction was completed, water (5 mL) was added into the jar and the mixture was milled for 3 min at 550 min^{-1}. The suspension was dropped into an Erlenmeyer flask containing acetone (80 mL), the balls were washed with water (2 × 3 mL), then with acetone (3 × 10 mL). The formed suspension was allowed to stand overnight at room temperature, with a cap, then the solid was removed by filtration (fritted glass, G3), washed with a mixture of 10:1 v/v water/acetone (10 mL), and then with acetone (3 × 10 mL). The solid was dried one day at 60–70 °C over KOH. 6^{I}-Monoazido-6^{I}-monodeoxy-β-CD (1.05–1.12 g, 90–97% yield) and TU-β-CD (0.94–1.18 g, 69–87% yield) were obtained as white solids.

General experimental procedure on 0.0001 mol scale (Table 1, entries 1, 5, 18–25).

The experimental procedure was similar to the one reported for the reaction at the 0.001 mol scale, except that Ts-β-CD (0.129 g), NaN_3 (0.020 g) or thiourea (TU, 0.023 g) were used.

Work-up and purification. As the reaction was completed, water (1 mL) was added into the jar and the mixture was milled for 3 min at 550 min^{-1}. The suspension was dropped into an Erlenmeyer containing acetone (50 mL), the balls were washed with water (2 × 0.5 mL), then with acetone (3 × 2 mL). The formed suspension was allowed to stand overnight at room temperature, with a cap, then the solid was removed by filtration (fritted glass, G3), washed with a mixture of 10:1 v/v water/acetone (2.5 mL), and then with acetone (3 × 5 mL). The solid was dried one day at 60–70 °C over KOH. 6^{I}-Monoazido-6^{I}-monodeoxy-β-CD (0.086–0.101 g, 74–87% yield) and TU-β-CD (0.101–1.121 g, 74–85 % yield) were obtained as white solids.

References

1 Mughal, A.; Chan, H. K.; Weaire, D.; Hutzler, S. *Physical Review E*, **2012**, *85*, 051305.
2 Burtseva, L.; Salas, B. V.; Werner, F.; Petranovskii, V. *Revista Mexicana de Física*, **2015**, *61*, 20–27.
3 Law, H.; Benito, J. M.; Garcia Fernandez, J. M.; Jicsinszky, L.; Crouzy, S.; Defaye, J. *J. Phys. Chem. B*, **2011**, *115*, 7524–7532.
4 Jicsinszky, L.; Caporaso, M.; Tuza, K.; Martina, K.; Gaudino, E. C.; Cravotto, G. *ACS Sustainable Chem. Eng.*, **2016**, *4*, 919–929.
